# Supplementary material for: Modeling the relationship between estimated fungicide use and disease-associated yield losses of soybean in the United States I: Foliar fungicides vs foliar diseases
Source: PLoS One. 2020 Jun 11;15(6):e0234390. doi: 10.1371/journal.pone.0234390 (PMC7289349; doi:10.1371/journal.pone.0234390)
Supplement: S1 Table — (DOCX) [file pone.0234390.s001.docx]

**Supplementary table 1.** Mixed-eﬀects modelling of the eﬀect of foliar fungicide use on soybean yield losses due to foliar diseases from soybean growing states in the northern region of the United States during 2005-2015 period. A = annual total fungicide use in MT and annual total production loss in 1,000 MT. B = annual total fungicide use in g/ha and annual yield loss in kg/ha. States included IL, IN, IA, KS, MI, MN, NE, ND, OH, PA, SD, and WI.

|  | A | | |  | B | | |
| --- | --- | --- | --- | --- | --- | --- | --- |
| Model name | Null model | Full model (L) | Full model (Q) |  | Null model | Full model (L) | Full model (Q) |
| **Fixed effect** | *a* ± SE | *a* ± SE | *a* ± SE |  | *a* ± SE | *a* ± SE | *a* ± SE |
| Intercept | 126.4 ± 34.9 | 126.4 ± 35.5 | 126.4 ± 34.8 |  | 93.4 ± 26.7 | 93.4 ± 24.9 | 93.4 ± 25.0 |
| Fungicide use | - | -24.9 ± 191.7 | 67.3 ± 197.4 |  | - | 218.9 ± 101.6 | 209.5 ± 103.8 |
| Fungicide use^2^ | - | - | -226.3 ± 139.2 |  | - | - | 43.2 ± 97.4 |
|  |  |  |  |  |  |  |  |
| **Random effects** | VC | VC | VC |  | VC | VC | VC |
| State | 10,297 | 10,671 | 10,134 |  | 7,118 | 6,404 | 6,408 |
| Year | 2,893 | 3,014 | 3,003 |  | 695 | 340 | 400 |
| Residuals | 12,672 | 12,709 | 12,587 |  | 7,313 | 7,388 | 7,403 |
|  |  |  |  |  |  |  |  |
| ***R^2^*_GLMM(_*_m_*_)_** | - | 0.000 | 0.016 |  | - | 0.025 | 0.024 |
| ***R^2^*_GLMM(_*_c_*_)_** | - | 0.519 | 0.519 |  | - | 0.490 | 0.492 |
| **AIC** | 1,669.6 | 1,671.6 | 1,670.9 |  | 1,593.3 | 1,591.0 | 1,592.9 |
| **BIC** | 1,681.1 | 1,686.0 | 1,688.2 |  | 1,604.8 | 1,605.5 | 1,610.2 |

L = linear; Q = quadratic; SE = standard error; VC = variance components. *R^2^*_GLMM(_*_m_*_)_ = generalized R^2^ for marginal model; *R^2^*_GLMM(_*_c_*_)_ = generalized R^2^ for conditional model; AIC = Akaike Information Criterion; BIC = Bayesian information criterion.
