# Supplementary material for: Modeling the relationship between estimated fungicide use and disease-associated yield losses of soybean in the United States I: Foliar fungicides vs foliar diseases
Source: PLoS One. 2020 Jun 11;15(6):e0234390. doi: 10.1371/journal.pone.0234390 (PMC7289349; doi:10.1371/journal.pone.0234390)
Supplement: S4 Table — (DOCX) [file pone.0234390.s004.docx]

**Supplementary table 4.** Mixed-eﬀects modelling of the eﬀect of foliar fungicide use on soybean production/yield from soybean growing states in the northern region of the United States during 2005-2015 period. A = annual total fungicide use in MT and annual total production in 1,000 MT. B = annual total fungicide use in g/ha and annual yield in kg/ha. States included IL, IN, IA, KS, MI, MN, NE, ND, OH, PA, SD, and WI.

|  | A | | |  | B | | |
| --- | --- | --- | --- | --- | --- | --- | --- |
| Model name | Null model | Full model (L) | Full model (Q) |  | Null model | Full model (L) | Full model (Q) |
| **Fixed effect** | *a* ± SE | *a* ± SE | *a* ± SE |  | *a* ± SE | *a* ± SE | *a* ± SE |
| Intercept | 5,780 ± 1,148 | 5,780 ± 1,087 | 5,780 ± 1,098 |  | 2,873 ± 137 | 2,873 ± 135 | 2,873 ± 137 |
| Fungicide use | - | 3,214 ± 1,222 | 2,794 ± 1,312 |  | - | 363 ± 340 | 304 ± 354 |
| Fungicide use^2^ | - | - | 697 ± 815 |  | - | - | 158 ± 287 |
|  |  |  |  |  |  |  |  |
| **Random effects** | VC | VC | VC |  | VC | VC | VC |
| State | 15,379,281 | 13,825,902 | 14,102,370 |  | 182,551 | 179,521 | 183,872 |
| Year | 374,863 | 288,246 | 293,148 |  | 34,426 | 30,680 | 32,163 |
| Residuals | 390,560 | 383,722 | 383,391 |  | 55,618 | 56,173 | 56,196 |
|  |  |  |  |  |  |  |  |
| ***R^2^*_GLMM(_*_m_*_)_** | - | 0.005 | 0.004 |  | - | 0.004 | 0.003 |
| ***R^2^*_GLMM(_*_c_*_)_** | - | 0.974 | 0.974 |  | - | 0.790 | 0.794 |
| **AIC** | 2,179.3 | 2,174.6 | 2,175.9 |  | 1,888.7 | 1,889.6 | 1,891.3 |
| **BIC** | 2,190.8 | 2,189.1 | 2,193.2 |  | 1,900.2 | 1,904.0 | 1,908.6 |

L = linear; Q = quadratic; SE = standard error; VC = variance components. *R^2^*_GLMM(_*_m_*_)_ = generalized R^2^ for marginal model; *R^2^*_GLMM(_*_c_*_)_ = generalized R^2^ for conditional model; AIC = Akaike Information Criterion; BIC = Bayesian information criterion.
