# Supplementary material for: Modeling the relationship between estimated fungicide use and disease-associated yield losses of soybean in the United States I: Foliar fungicides vs foliar diseases
Source: PLoS One. 2020 Jun 11;15(6):e0234390. doi: 10.1371/journal.pone.0234390 (PMC7289349; doi:10.1371/journal.pone.0234390)
Supplement: S5 Table — (DOCX) [file pone.0234390.s005.docx]

**Supplementary table 5.** Mixed-eﬀects modelling of the eﬀect of foliar fungicide use on soybean production/yield from soybean growing states in the southern region of the United States during 2005-2015 period. A = annual total fungicide use in MT and annual total production in 1,000 MT. B = annual total fungicide use in g/ha and annual yield in kg/ha. States included AL, AR, DE, FL, GA, KY, LA, MD, MS, MO, NC, OK, SC, TN, TX, and VA.

|  | A | | |  | B | | |
| --- | --- | --- | --- | --- | --- | --- | --- |
| Model name | Null model | Full model (L) | Full model (Q) |  | Null model | Full model (L) | Full model (Q) |
| **Fixed effect** | *a* ± SE | *a* ± SE | *a* ± SE |  | *a* ± SE | *a* ± SE | *a* ± SE |
| Intercept | 1,183 ± 368 | 1,183 ± 352 | 1,183 ± 341 |  | 2,330 ± 118 | 2,330 ± 118 | 2,330 ± 116 |
| Fungicide use | - | 2,117 ± 364 | 2,618 ± 394 |  | - | 179 ± 405 | 240 ± 409 |
| Fungicide use^2^ | - | - | -1,055 ± 351 |  | - | - | -385 ± 358 |
|  |  |  |  |  |  |  |  |
| **Random effects** | VC | VC | VC |  | VC | VC | VC |
| State | 2,088,271 | 1,921,807 | 1,800,533 |  | 71,678 | 72,254 | 74,091 |
| Year | 45,460 | 33,088 | 33,030 |  | 96,347 | 95,953 | 90,785 |
| Residuals | 85,293 | 71,890 | 68,683 |  | 101,391 | 101,885 | 101,914 |
|  |  |  |  |  |  |  |  |
| ***R^2^*_GLMM(_*_m_*_)_** | - | 0.012 | 0.023 |  | - | 0.001 | 0.004 |
| ***R^2^*_GLMM(_*_c_*_)_** | - | 0.965 | 0.965 |  | - | 0.623 | 0.620 |
| **AIC** | 2,616.9 | 2,587.9 | 2,580.9 |  | 2,598.8 | 2,600.6 | 2,601.4 |
| **BIC** | 2,629.6 | 2,603.7 | 2,599.9 |  | 2,611.4 | 2,616.4 | 2,620.4 |

L = linear; Q = quadratic; SE = standard error; VC = variance components. *R^2^*GLMM(*m*) = generalized R^2^ for marginal model; *R^2^*GLMM(*c*) = generalized R^2^ for conditional model; AIC = Akaike Information Criterion; BIC = Bayesian information criterion.
